# Genetic polymorphisms in folate-metabolizing genes associated with gastric cancer prognosis in northwest China subjects

**DOI:** 10.7150/jca.46978

**Published:** 2020-09-13

**Authors:** Lijuan Yuan, Ziyu Liu, Gang Wei, Ping Yang, Xi E Hu, Falin Qu, Jianguo Lu, Xianli He, Guoqiang Bao

**Affiliations:** 1 Department of General Surgery, Tangdu Hospital, The Air Force Military Medical University, Xi'an, 710038, China.; 2 Department of Microbiology, The Air Force Military Medical University, Xi'an, 710032, China.

**Keywords:** folate metabolism, *MTRR*, * MTHFR*, *MTR*, polymorphism, gastric cancer prognosis

## Abstract

Influence of folate metabolism has long been studied in cancer and copies evidences have suggested that the key genes involved were correlated with GC risk and prognosis. However, their genetically association and contribution for GC prognosis are still elusive. To evaluate the effect of folate metabolism related genes polymorphisms on the prognosis of gastric cancer (GC), the genotype of seven single nucleotide polymorphisms (SNPs) of three genes were selected and genotyped in a cohort of 664 GC patients, including genes of Methylenetetrahydrofolate reductase (*MTHFR*), Methionine synthase reductase (*MTRR*), and Methionine synthase (*MTR*). Kaplan-Meier Curve, long-rank tests and multivariate Cox proportional hazard model were used for prognosis analysis. The results demonstrated that TT or CT/TT genotypes of SNP rs1532268 in *MTRR* gene coding region are significantly associated with a poorer overall survival (OS) when compared with CC genotype (HR=2.340, 95% CI: 1.240-4.414, *p*=0.009; or HR=1.502, 95% CI: 1.083-2.085, *p*=0.015, respectively). Furthermore, comparing to that of the CC genotype, the detrimental effect of rs1532268 TT genotype was also evident in the special subgroups of GC patients, especially in patients with BMI<24 and H. pylori infection. Moreover, significant association between increased relapse and TT genotype of rs1532268 was also observed in patients who are females, BMI<24 and without chemotherapy. In addition, the joint analysis demonstrated that integration of rs1532268 genotypes and BMI, H. pylori infection status, clinical stage and tumor site may significantly improve the predictive abilities for predicting OS of GC patients. In conclusion, it suggested that the *MTRR* rs1532268 polymorphism is significantly associated with clinical outcomes of GC patients, especially in those with lower BMI (BMI<24) or positive H. pylori infection status, which warrants further validation. And the polymorphism of *MTRR* rs1532268 may be a potential prognostic factor for GC patients.

## Introduction

Gastric cancer is the third deadliest cancer in the world and the absolute number of cases is increasing every year due to aging and growing of high-risk populations 1. Geographically, about 43% of total global cases are concentrated in China 2 and its mortality remains the third among all human cancers in China 3. The development of gastric cancer (GC) represents a complex interaction of host factors with infections agents and environmental factors 4**.** Despite recent important developments in our understanding of the pathophysiology of GC, patients diagnosed with this disease still have a poor prognosis, with a 5-year survival rate <25% 5.

There are still reduced therapeutic options for GC patients and the survival and prognosis of GC patients still depend on the stage of the tumor at the time of diagnosis. Recently, genomic analyses of gastric tumors have emphasized their molecular heterogeneity 6. The distinction of gastric cancer molecular subtypes may be a key to identify novel therapeutic targets, to guide early diagnosis strategies, predict patient outcome, and response to therapy 7, 8.

It has been suggested that dysfunctions of folate-mediated one-carbon metabolism (FOCM) could contribute to carcinogenesis, which is a key pathway essential for the processes of DNA synthesis, methylation and repair 9, 10. Furthermore, it may highlight therapeutic targets for gastrointestinal cancer 11. The enzymes, including Methylenetetrahydrofolate reductase (MTHFR), Methionine synthase (MTR) and Methionine synthase reductase (MTRR) are crucial components in FOCM, to catalyze S-adenosylmethionine (SAM) synthesis from folate uptake. MTHFR is crucial rate-limiting step for FOCM, carrying out an irreversible conversion of 5,10-methylene-tetrahydrofolate (5,10-MTHF) to 5-methyl-tetrahydrofolate (5-MTHF) 12. MTR catalyzes the re-methylation of homocysteine to methionine, which is a precursor of SAM 13. And MTRR is a flavor-protein maintains the activity of MTR 14. The genetic variants of these genes may influence enzyme activity and folate status, which may modulate gastric cancer development and progress 9, 12, 15.

Accumulating evidences have supported that the functional polymorphisms of the genes of *MTHFR, MTR* and *MTRR* may affect the risk of GC 16-24, but the results are variable. Recently, the effects of genomic polymorphisms in FOCM related genes on survival of gastric cancer patients were studied 25, 26, however, it still need more evidences to unveil the detailed association and enable better prognosis. Herein, the effects of seven SNPs of these three folate metabolizing genes on the clinical outcomes of 664 Chinese people were assessed. Additionally, we performed analyses stratified by BMI status to address the possibility that the lifestyle factor may modify the effect on the genetic polymorphism of GC clinical outcomes, besides age, sex, tumor diameter, H. pylori infection status, clinical stage and chemotherapy.

## Materials and Methods

### Study subjects

681 pathologically confirmed incident GC cases were enrolled from the Tangdu hospital (between November, 2007 and October, 2012) and Xijing Hospital (between October, 2006 to May, 2009) in Shaanxi province. All GC cases received surgical resection and had no previous history of other cancers or any preoperative anticancer treatment or blood transfusion within 3 months before surgery. There were no age, sex, or disease stage restrictions for case recruitment. Socio-demographic and clinical data were collected during recruitment. Clinical staging of GC tumors was done according to the WHO standard. Finally, 664 patients with resected gastric adenocarcinoma were included in the present study for prognostic analysis. The present study protocol was approved by the Institutional Review Board of Air Force Military Medical University. The procedures were performed according to the approved guidelines and the 1964 Helsinki Declaration and its later amendments or comparable ethical standards. Informed consent was obtained from each participant included in the study.

### Demographic and clinical data

Demographic and clinical data were collected through in-person interviews at the initial visit or follow up in the clinics, medical records, or consultation with treating physicians, including age, sex, ethnicity, residential region, time of diagnosis, time of surgery and/or adjuvant chemotherapy (ACT), time of relapse and/or death, tumor stage, and treatment protocol. Clinical stage 0 and I were sorted as early stage group, while clinical stage II and III were considered as middle stage group, clinical IV were considered as late stage group. Cases were followed for survival status and chemotherapy data every 6 months. The latest follow-up data in this analysis was obtained in October 2014. Overall survival (OS) was defined as the time from surgery to GC-specific death. Relapse-free survival (RFS) was defined as the time from surgery to the date of the first recurrence or distant metastasis of GC. Patients alive at the last follow-up were censored.

### Genotyping

Peripheral blood samples from GC patients were drawn in to coded sodium citrate anticoagulant tubes and were centrifuged within 30 min by the investigators. The E.Z.N.A. Blood DNA Midi Kit (Omega Bio-Tek, Norcross, GA, USA) was used for genomic DNA extraction. All the genomic DNA was aliquoted and stored at -80 °C for future analysis.

The candidate functional SNPs of the folate metabolism related genes *MTHFR, MTR* and *MTRR* were performed according to a set of web-based SNP selection tools (https://manticore.niehs.nih.gov/snpinfo/snpfunc.html)^27^. Finally, seven functional SNPs were selected, including *MTHFR* rs2274976 (C>T), rs1801133 (G>A); *MTR* rs1805087 (A>G), rs2853522 (A>C); *MTRR* rs1801394 (A>G), rs1532268 (C>T), rs162036 (A>G). Genotyping of seven candidate SNPs was performed using Agena MassARRAY RS1000 system according to the standard protocol (Applied Biosystems, Foster City, CA, USA). Internal quality controls were used to ensure genotyping accuracy.

### Statistical analysis

Statistics analyses were carried out using the IBM SPSS Statistics 20.0 software (IBM). Kaplan-Meier curves and log-rank tests were also used to evaluate effect of the individual SNPs on OS and RFS. Cox proportional hazard regression model was applied to assess the effect of individual SNP and patients' characteristics on OS or RFS. Hazard ratios (HRs) and 95% confidence intervals (CIs) were estimated with adjustment for age, gender, BMI, H. pylori infection status, clinical stage, tumor diameter, and chemotherapy status. All statistical tests were two-sided, with *p* < 0.05 as the boundary value.

## Results

### Association of polymorphisms and clinical outcome

The clinical characteristics of 664 GC patients were summarized in **Table 1.** At latest follow-up, 287 patients developed relapse and 226 died. Significant OS and RFS of patients were observed among subgroup of tumor size, clinical stage and chemotherapy condition by univariate Cox regression analysis (*p* <0.05), respectively (**Table 1**).

**Figure 1:**
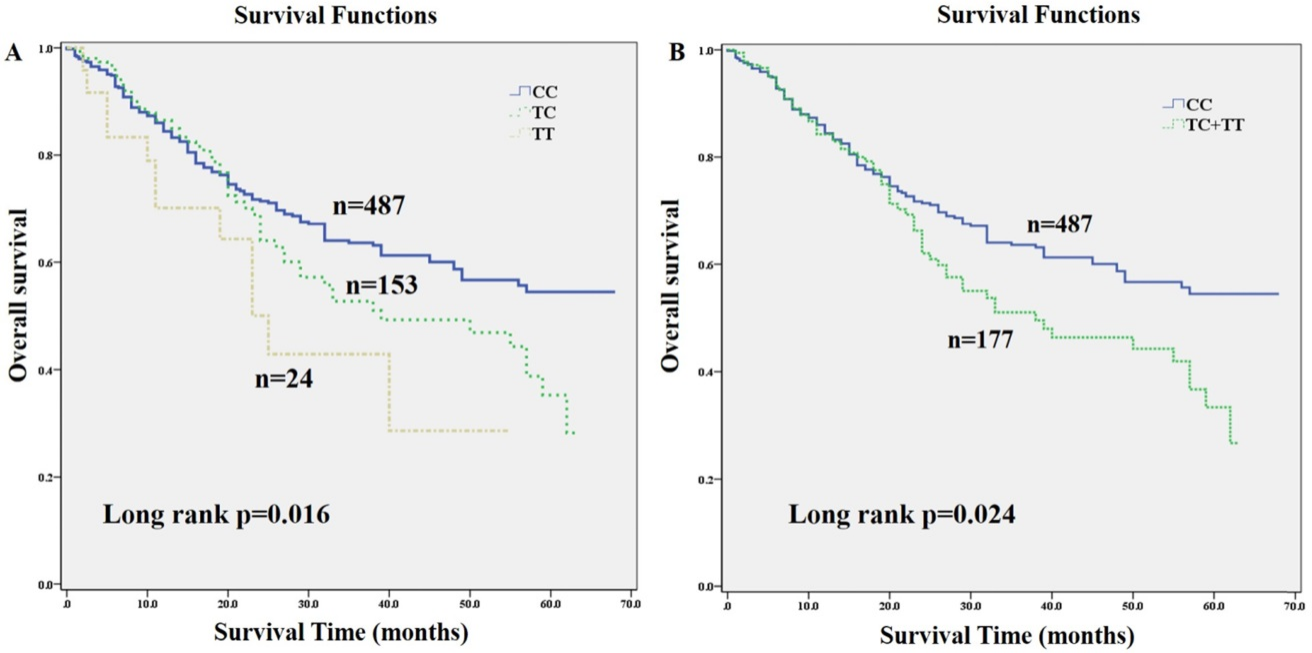
Kaplan-Meier estimates of overall survival (OS) for gastric cancer (GC) patients stratified by genetic variants of *MTRR* gene. OS of GC patients stratified by SNP rs1532268. (**A**) Overall survival of *MTRR* rs1532268 co-dominant genotypes in GC patients; (**B**) overall survival of *MTRR* rs1532268 dominant genotypes in GC patients.

The association of three folate metabolizing genes SNP genotypes with GC clinical outcome were assessed using multivariate Cox regression analysis with adjustment for age, gender, tumor diameter, BMI status, H. pylori infection status, clinical stage and chemotherapy (Table 2). The results showed that SNP rs1532268 polymorphism was significantly associated with OS of GC patients. Compared to patients with CC genotype, patients with TT genotype or combined TT/CT genotypes had significantly higher death risk (HR=2.34, 95% CI: 1.240-4.414, *p*=0.009; HR=1.502, 95% CI: 1.083-2.085, *p*=0.015), respectively. In addition, Kaplan-Meier curves analysis also provided a strong association with OS. The median OS time was 47 months in patients with the CC genotype, 39 months in patients with CT genotype, 25 months in patients with TT genotype. Patients carrying TT or combined TT/CT genotypes of rs1532268 had worse OS than did those with CC genotype (*p*=0.016; *p*=0.024) (**Fig. 1**), respectively, which also indicated that the rs1532268 polymorphism played prognostic role in GC. However, negative results were obtained for the other SNPs involved in this study using multivariate Cox regression analysis.

### Stratified analysis on association of *MTRR* rs1532268 polymorphisms with clinical outcome by host variables

Stratified analyses were conducted to evaluate the associations between genotypes of *MTRR* rs1532268 and clinical outcome by age, gender, clinical stage, H. pylori infection status, BMI, tumor diameter, tumor site and ACT (**Table 3**). The significant detrimental effects conferred by rs1532268 TT genotype was more prominent in special subgroups. Compared with CC genotype, the significant increased death risk associated with TT genotype of rs1532268 was observed in patients in age <60 years (HR=3.064, 95% CI: 1.400-6.704), females (HR=6.975, 95% CI: 1.475-32.981), with positive H. pylori infection status (HR=3.169, 95% CI: 1.497-6.712), negative ACT status (HR=4.249, 95% CI: 1.252-14.422), middle stage GC (clinical stage II/III) (HR=2.245, 95% CI: 1.118-4.509), and BMI<24 (HR=3.217, 95% CI: 1.672-6.190). Moreover, TC and TT genotypes of rs1532268 showed association with increased death risk in patients with non-cardia GC (HR=1.616, 95% CI: 1.077-2.516; HR=2.367, 95% CI: 1.063-5.270), respectively, when compared with CC genotype. Furthermore, the significant increased relapse risk associated with TT genotype of rs1532268 was also observed in female patients (HR=4.827, 95% CI: 1.083-21.523), and patients with BMI<24 (HR=2.265, 95% CI: 1.221-4.200), negative ACT status (HR=4.674, 95% CI: 1.390-15.719), cardia GC (HR=3.170, 95% CI: 1.042-9.638), when compared with CC genotype.

### Joint effect between rs1532268 genotypes and H. pylori infection, BMI status, clinical stage, tumor site and chemotherapy on OS

A joint analysis was performed to assess the potential modulating effect of rs1532268 polymorphism on the clinical characteristics associated with OS of GC patients. Comparing with patients carrying rs1532268 CC genotypes and without H. pylori infection, those with TT genotype and H. pylori infection had a significantly increase death risk (HR=2.361, 95% CI: 1.086-5.134). The similar results were also observed in patients with rs1532268 TT genotype and BMI <24 (HR=2.691, 95% CI: 1.414-5.122). Moreover, compared with patients carrying CC genotype and non-cardia type, patients with TC genotype and non-cardia type (HR=1.583, 95% CI: 1.041-2.406), TT genotype and non-cardia type (HR=2.233, 95% CI: 1.014-4.917), CC genotype and cardia type (HR=2.12, 95% CI: 1.402-3.207) showed increased death risk. In addition, compared with patients with CC genotype and in early stage, patients with CC genotype and in middle stage (HR=3.951, 95% CI: 1.864-8.371), CC genotype and in late stage (HR=16.234, 95% CI: 7.033-37.476), TC genotype and in middle stage (HR=6.034, 95% CI: 2.706-13.452), TC genotype and in late stage (HR=14.791, 95% CI: 5.057-43.262), TT genotype and in middle stage (HR=8.835, 95% CI: 3.307-23.605), TT genotype and in late stage (HR=33.751, 95% CI: 6.861-166.040) also showed increased death risk. However, negative results were observed in the integration of rs1532268 genotypes and chemotherapy status. In conclusion, these results provide the potential additional predictive abilities of rs1532268 polymorphism and the clinical characters in predicting GC OS.

## Discussion

More attention has been paid on the folate metabolism for its important role in cancer 10. *MTHFR, MTR* and *MTRR* genes play key roles in folate metabolism pathway and were most examined in cancer risk and prognosis. In present study, the effects of polymorphisms in these genes on prognosis of GC patients were investigated. The results demonstrated that TT or CT/TT genotypes of SNP rs1532268 in *MTRR* coding region are significantly associated with a poorer OS in a set of 664 GC patients when compared with CC genotypes. Furthermore, comparing to that of the CC genotype, the detrimental effect of rs1532268 TT genotype was also evident in the subgroups of GC patients. The rs1532268 TT genotype was associated with increased death risk of GC patients in age <60 years, females, BMI<24, middle stage GC (clinical stage II/III), with H. pylori infection and without chemotherapy. Moreover, significant association between increased relapse and TT genotype of rs1532268 was also observed in patients who are females, BMI<24 and without chemotherapy.

Copies evidences proved that *MTRR* polymorphisms were associated with risk and prognosis of GC. But most reports focus on the *MTRR* rs1801394 polymorphisms, which displayed a protective effect on GC among Chinese population 25, 26. Our knowledge on the association of cancer and *MTRR* rs1532268 polymorphism is very limited. It has been reported that *MTRR* rs1532268 polymorphism was associated with increased risk of prostate cancer 28, while other studies did not reveal any obviously significant differences of *MTRR* rs1532268 polymorphisms among other cancers. Our finding indicated that patients with *MTRR* rs1532268 CT/TT genotypes played harmful role on GC prognosis.

MTRR is a flavoprotein that maintains the activity of MTR 14, which catalyzed the remethylation of homocysteine to produce methionine, functioning as a precursor for the universal methyl group donor S-adenosylmethionine. The polymorphism of *MTRR* rs1532268, causing a serine to leucine switch in protein sequence, may impact MTRR enzymatic activity. Definitely positive relationship between polymorphisms of rs1532268 and gastrointestinal stomal tumor has been noted (https://www.snpedia.com/index.php/Special:FormEdit/ClinVar_Disease/Gastrointestinal_stromal_tumor), and we found that *MTRR* rs1532268 CT/TT genotype showed a comparatively worse OS of GC patients in this study, especially the TT genotype was associated with increased death risk in middle stage GC patients. The reason may be that the *MTRR* TT/CT genotype might reduce the affinity of MTRR for MTR and less efficient reactivation 29, leading to increased homocysteine 30. Therefore, the less remethylation of homocysteine may generate less methionine for DNA methylation. It is proposed that the reduced methylation on promoters of tumor genes would strength the GC cell growth and invasiveness.

Furthermore, the current study demonstrated that the SNP rs1532268 affect OS and RFS of GC patients more prominent in specific subgroup patients. Obesity is a major health issue and a risk factor for cancer prognosis 31. BMI ≥24 was used to designate over-weight in Chinese, and rs1532268 TT genotype had poor prognosis in patients with low BMI (BMI<24), while the effect of BMI on gastric cancer is still inconsistent 32-34. H. pylori infection is another important factor in GC risk 35, significant relationship between rs1532268 polymorphism and clinical outcomes was also observed in the patients with H. pylori infection. And for females it also showed worse OS and RFS, who was considered as low gastric cancer risk. In addition, integration of rs1532268 genotypes and clinical characters may improve the predictive abilities for predicting OS of GC patients. Therefore, the genetic factor rs1532268 polymorphism showed its interactions among patients with different HP infection status and life style factors and its promising role in modulating tumor progression.

Overall, our data strongly suggest that rs1532268 of *MTRR* involved in folate metabolism pathway had a significant effect on the clinical outcome of GC patients in a Chinese population, especially for patients with lower BMI or positive HP infection status. The present study has potential clinical significance in helping to refine therapeutic decisions in treatment of GC. Moreover, since our study was restricted to Han Chinese, we cannot rule out the generalizability issue. Future studies in larger populations and other ethnics are warranted.

## Figures and Tables

**Table 1:** Distribution of patients' characteristics and prognosis analysis

Variables		OS			RFS		
		Total/Death	HR (95%CI)	*p*^a^	Total/Relapse	HR (95%CI)	*p*^a^
Age	<60	375/122	1.000		375/159	1.000	
	≥60	289/104	1.185 (0.912-1.539)	0.204	289/128	1.064 (0.843-1.343)	0.601
Gender	male	512/168	1.000		512/213	1.000	
	female	152/58	1.178 (0.874-1.588)	0.283	152/74	1.214 (0.932-1.581)	0.151
BMI^b^	<24	416/135	1.000		416/170	1.000	
	≥24	163/46	0.858 (0.614-1.199)	0.370	163/60	0.881 (0.656-1.182)	0.398
H. pylori^b^	No	187/55	1.000		187/81	1.000	
	Yes	392/126	1.002 (0.728-1.38)	0.989	392/149	0.851 (0.648-1.117)	0.245
Tumor diameter^b^	<5	377/93	1.000		377/128	1.000	
	≥5	273/128	2.071 (1.586-2.705)	**<0.001**	273/153	1.968 (1.556-2.489)	**<0.001**
Clinical stages^b^	0	14/2	1.000		14/2	1.000	
	Ⅰ	126/9	0.478 (0.103-2.215)	0.345	126/13	0.669 (0.151-2.965)	0.597
	Ⅱ	316/102	2.659 (0.655-10.789)	0.171	316/129	3.418 (0.845-13.819)	0.085
	Ⅲ	148/70	5.986 (1.464-24.47)	**0.013**	148/95	8.789 (2.162-35.734)	**0.002**
	Ⅳ	55/41	12.394 (2.986-51.445)	**<0.001**	55/45	14.937 (3.61-61.802)	**<0.001**
**ACT**^b^	No	233/58	1.000		233/63	1.000	
	Yes	430/168	1.515 (1.124-2.043)	**0.006**	430/224	2.029 (1.534-2.684)	**<0.001**

Note: HR: hazard ratio; CI: confidence interval; ACT, adjuvant chemotherapy; ^a^: univariate analysis by COX proportional hazard regression model; ^b^: Patient numbers may not add up to 100% of available subjects because of missing clinical data.

**Table 2:** Genotypes of *MTHFR, MTR, MTRR* genes polymorphism with clinical outcome of gastric cancer patients

SNP ID	Genotype	Total/Events	OS				Total/Events	RFS			
			*p*^c^	MST^b^	HR (95%CI)	*p*^a^		*p*^c^	MST	HR (95%CI)	*p*^a^
rs2274976	CC	588/202		57	1.000		588/255		34	1.000	
	TC	73/22	**0.001**	47^b^	0.614 (0.352-1.072)	0.086	73/30	**0.004**	51	0.641 (0.393-1.045)	0.074
	TT	3/2		12	4.882 (0.67-35.589)	0.118	3/2		4	4.014 (0.543-29.688)	0.173
	Dominant	76/24	0.528	46^ b^	0.655 (0.382-1.123)	0.124	76/32	0.844	51	0.672 (0.417-1.083)	0.103
rs1801133	AA	189/62		57	1.000		189/78		34	1.000	
	GA	318/96	**0.018**	47^ b^	1.015 (0.715-1.441)	0.934	318/128	**0.036**	55	1.019 (0.748-1.389)	0.904
	GG	154/67		32	1.189 (0.791-1.786)	0.405	154/79		22	1.112 (0.77-1.604)	0.572
	Dominant	472/163	0.7	62	1.07 (0.773-1.48)	0.683	472/207	0.619	38	1.048 (0.785-1.399)	0.751
rs1805087	AA	562/194		57	1.000		562/240		37	1.000	
	GA	98/29	0.221	48^ b^	0.763 (0.485-1.201)	0.242	98/43	**0.028**	32	1.029 (0.712-1.487)	0.881
	GG	4/3		19	0.848 (0.194-3.703)	0.826	4/4		5	3.516 (0.807-15.325)	0.094
	Dominant	102/32	0.334	47^ b^	0.769 (0.498-1.189)	0.238	102/47	0.945	28	1.075 (0.75-1.539)	0.695
rs2853522	CC	218/82		49	1.000		218/102		26	1.000	
	AC	322/106	0.455	62	0.914 (0.658-1.270)	0.593	322/141	0.321	38	0.961 (0.719-1.283)	0.787
	AA	123/38		57	0.784 (0.499-1.232)	0.291	123/44		40^b^	0.7 (0.461-1.063)	0.094
	Dominant	445/144	0.218	62	0.879 (0.643-1.201)	0.418	445/185	0.280	42	0.89 (0.675-1.174)	0.410
rs1532268	CC	487/156		47^b^	1.000		487/208		46	1.000	
	TC	153/58	**0.016**	39	1.386 (0.975-1.97)	0.069	153/66	0.257	29	1.084 (0.788-1.49)	0.621
	TT	24/12		25	**2.340 (1.240-4.414)**	**0.009**	24/13		16	1.692 (0.927-3.088)	0.087
	Dominant	177/70	**0.024**	38	**1.502 (1.083-2.085)**	**0.015**	177/79	0.435	26	1.158 (0.86-1.559)	0.333
rs162036	AA	466/164		57	1.000		466/209		31	1.000	
	GA	181/58	0.654	45^ b^	0.929 (0.666-1.296)	0.666	181/75	0.171	42	0.944 (0.706-1.263)	0.700
	GG	16/4		56	0.682 (0.167-2.786)	0.594	16/3		46^ b^	0.24 (0.033-1.722)	0.156
	Dominant	197/62	0.522	45^ b^	0.916 (0.66-1.271)	0.600	197/78	0.339	40^ b^	0.903 (0.677-1.206)	0.491
rs1801394	AA	358/122	0.997	57	1.000		258/155		35	1.000	
	GA	260/90		59	1.014 (0.744-1.383)	0.930	260/118	0.512	33	1.261 (0.962-1.654)	0.093
	GG	45/14		50	0.854 (0.434-1.679)	0.647	45/14		40^ b^	0.763 (0.395-1.474)	0.421
	Dominant	305/104	0.946	57	0.994 (0.735-1.345)	0.971	305/132	0.908	37	1.195 (0.917-1.559)	0.188

^a^: adjusted by age, gender, BMI, H. pylori infection status, clinical stage, tumor diameter, and chemotherapy status; ^b^:Mean survival time was provided when MST could not be calculated; ^c^: Log-rank *p;* HR: hazard ratio; CI: confidence interval; MST, median survival time.

**Table 3:** Stratified analysis of the *MTRR* rs1532268 polymorphism with gastric cancer OS and RFS

		Genotype	Total/ Event	OS		Total/ Event	RFS	
				HR (95%CI)	*p*^a^		HR (95%CI)	*p*^a^
Age	<60	CC	277/83	1.000		277/115	1.000	
		TC	83/31	1.559 (0.958-2.538)	0.074	83/36	1.223 (0.796-1.878)	0.359
		TT	15/8	**3.064 (1.4-6.704)**	**0.005**	15/8	1.741 (0.816-3.715)	0.152
	≥60	CC	210/73	1.000		210/93	1.000	
		TC	70/27	1.396 (0.825-2.362)	0.214	70/30	1.018 (0.623-1.662)	0.944
		TT	9/4	1.752 (0.533-5.759)	0.355	9/5	1.782 (0.633-5.018)	0.274
Gender	female	CC	115/40	1.000		115/57	1.000	
		TC	34/15	2.185 (0.992-4.814)	0.052	34/14	1.33 (0.635-2.787)	0.450
		TT	3/3	6.975 (1.475-32.981)	**0.014**	**3/3**	**4.827 (1.083-21.523)**	**0.039**
	male	CC	372/116	1.000		372/151	1.000	
		TC	119/43	1.239 (0.822-1.867)	0.307	119/52	1.09 (0.758-1.567)	0.642
		TT	21/9	1.898 (0.942-3.824)	0.073	21/10	1.47 (0.761-2.841)	0.252
H. pylori	no	CC	139/39	1.000		139/61	1.000	
		TC	41/13	1.31 (0.659-2.605)	0.442	41/17	1.088 (0.611-1.938)	0.774
		TT	7/3	1.328 (0.395-4.464)	0.646	7/3	1.245 (0.374-4.142)	0.721
	Yes	CC	284/85	1.000		284/104	1.000	
		TC	92/33	1.383 (0.913-2.094)	0.126	92/36	1.088 (0.739-1.602)	0.670
		TT	16/8	**3.169 (1.497-6.712)**	**0.003**	16/9	**2.229 (1.104-4.499)**	**0.025**
BMI	BMI≥24	CC	120/34	1.000		120/45	1.000	
		TC	40/12	1.343 (0.665-2.71)	0.411	40/15	1.183 (0.638-2.193)	0.594
		TT	3/0	NA	0.972	3/0	NA	0.976
	BMI<24	CC	303/90	1.000		303/120	1.000	
		TC	93/34	1.491 (0.983-2.261)	0.060	93/38	1.139 (0.779-1.666)	0.503
		TT	20/11	3.217 (1.672-6.190)	**<0.001**	20/12	**2.265 (1.221-4.200)**	**0.009**
Tumor diameter	< 5 cm	CC	278/61	1.000		278/90	1.000	
		TC	84/26	1.573 (0.934-2.65)	0.088	84/31	1.136 (0.72-1.793)	0.583
		TT	15/6	2.417 (1.012-5.773)	**0.047**	15/7	1.722 (0.778-3.813)	0.180
	≥5 cm	CC	201/92	1.000		201/114	1.000	
		TC	63/30	1.258 (0.770-2.054)	0.359	63/33	1.092 (0.688-1.732)	0.709
		TT	9/6	2.818 (1.085-7.321)	**0.033**	9/6	2.013 (0.789-5.133)	0.143
Clinical stage	early	CC	108/10	1.000		108/14	1.000	
		TC	28/1	0.491 (0.055-1.372)	0.524	28/1	0.37 (0.047-2.944)	0.347
		TT	4/0	NA	0.991	4/0	NA	0.986
	middle	CC	335/113	1.000		335/158	1.000	
		TC	112/49	1.48 (0.999-2.193)	0.050	112/55	1.084 (0.762-1.543)	0.652
		TT	17/10	2.245 (1.118-4.509)	**0.023**	17/11	1.702 (0.885-3.274)	0.111
	late	CC	42/32	1.000		42/35	1.000	
		TC	10/7	0.814 (0.309-2.146)	0.678	10/8	1.08 (0.453-2.575)	0.863
		TT	3/2	2.710 (0.522-14.078)	0.236	3/2	2.363 (0.475-11.758)	0.294
ACT	negative	CC	186/44	1.000		186/51	1.000	
		TC	41/11	1.984 (0.929-4.234)	0.077	41/9	1.205 (0.530-2.737)	0.656
		TT	6/3	4.249 (1.252-14.422)	**0.020**	6/3	4.674 (1.390-15.719)	**0.013**
	positive	CC	301/112	1.000		301/157	1.000	
		TC	111/47	1.267 (0.852-1.885)	0.243	111/57	1.072 (0.758-1.517)	0.693
		TT	18/9	2 (0.948-4.221)	0.069	18/10	1.344 (0.673-2.685)	0.403
Tumor site	Cardia	CC	87/35	1.000		87/43	1.000	
		TC	34/12	1.02 (0.507-2.050)	0.956	34/15	0.797 (0.428-1.483)	0.474
		TT	4/3	2.187 (0.589-8.12)	0.242	4/4	3.170 (1.042-9.638)	**0.042**
	Non-cardia	CC	312/80	1.000		312/112	1.000	
		TC	95/33	1.646 (1.077-2.516)	**0.021**	95/37	1.268 (0.862-1.865)	0.228
		TT	18/7	2.367 (1.063-5.270)	**0.035**	18/7	1.361 (0.621-2.982)	0.441

^a^: adjusted by age, gender, BMI, H. pylori infection status, clinical stage, tumor diameter, and chemotherapy status; NA: the corresponding value could not be calculated; HR: hazard ratio; CI, confidence interval; ACT, adjuvant chemotherapy.

**Table 4:** Joint effect of rs1532268 genotypes and HP infection, BMI status, clinical stage, tumor site and chemotherapy on OS

Variables	HR (95%CI)	*p*^a^
**Genotype with/without H. pylori**		
CC+ without H. pylori infection	1.000	
CC+ with H. pylori infection	0.869 (0.589-1.283)	0.480
TC+ without H. pylori infection	1.316 (0.684-2.531)	0.411
TC+ with H. pylori infection	1.178 (0.732-1.894)	0.500
TT+ without H. pylori infection	1.331 (0.408-4.346)	0.635
TT+ with H. pylori infection	2.361 (1.086-5.134)	**0.030**
**Genotype + BMI**		
CC+BMI<24	1.000	
CC+BMI≥24	1.097 (0.725-1.658)	0.662
TC+BMI<24	1.414 (0.937-2.135)	0.099
TC+BMI≥24	1.280 (0.692-2.365)	0.431
TT+BMI<24	2.691 (1.414-5.122)	**0.003**
TT+BMI≥24	NA	0.959
**Genotype stage**		
CC+ early stage	1.000	
CC+ middle stage	3.951 (1.864-8.371)	**<0.001**
CC+ late stage	16.234 (7.033-37.476)	**<0.001**
TC+ early stage	0.594 (0.074-4.760)	0.624
TC+ middle stage	6.034 (2.706-13.452)	**<0.001**
TC+ late stage	14.791 (5.057-43.262)	**<0.001**
TT+ early stage	NA	0.959
TT+ middle stage	8.835 (3.307-23.605)	**<0.001**
TT+ late stage	33.751 (6.861-166.040)	**<0.001**
**Genotype with/without chemotherapy**		
CC+ without chemotherapy	1.000	
CC+ with chemotherapy	0.884 (0.597-1.307)	0.535
TC+ without chemotherapy	1.843 (0.889-3.821)	0.100
TC+ with chemotherapy	1.083 (0.683-1.718)	0.735
TT+ without chemotherapy	2.967 (0.909-9.686)	0.072
TT + with chemotherapy	1.67 (0.774-3.607)	0.191
**Genotype with/without cardia**		
CC+ Non-cardia	1.000	
CC+ cardia	2.120 (1.402-3.207)	**<0.001**
TC+ Non-cardia	1.583 (1.041-2.406)	**0.032**
TC+ cardia	1.822 (0.971-3.420)	0.062
TT+ Non-cardia	2.233 (1.014-4.917)	**0.046**
TT+ cardia	3.100 (0.958-10.033)	0.059

^a^: adjusted by age, gender, BMI, H. pylori infection status, clinical stage, tumor diameter, and chemotherapy status. NA: the corresponding value could not be calculated.
